# Gynæcological and Obstetrical Society of Baltimore

**Published:** 1889-02

**Authors:** 


### *GYNÆCOLOGICAL AND OBSTETRICAL SOCIETY OF BALTIMORE.*

*Regular Meeting, October 9, 1888.*

THE PRESIDENT IN THE CHAIR.

DR. H. P. C. WILSON reported a case of

### LAPAROTOMY FOR FIBRO-SARCOMA OF THE OVARY.

Mrs. J., aged 47. Mother of 5 children —youngest 15 years old. Had hard, lingering labors. No instrument used. During her last pregnancy, she fell 18 feet out of a window—head-foremost—with such force as to burst open her corsets, and yet she did not miscarry.

In September, 1887, she first noticed a lump in the lower abdomen, on the right side. In January, 1888, she had rapid abdominal enlargement and swelling in both lower limbs. The limbs were punctured with lancet; water ran out, and abdomen and limbs decreased in size. In July, 1888, she was tapped, and 5 gallons of fluid drawn off.

She was brought to me at the Union Protestant Infirmary the latter part of September, 1888.

On examination I found her abdomen greatly enlarged with peritonitic fluid, and also a hard tumor growing from the right ovary—larger than a child's head—and not connected with the uterus, but apparently floating free in the peritoneal fluid. Uterus in place, and measuring three inches. Heart sound, and all other organs acting well. Nothing to account for the dropsy, but the solid tumor of the ovary.

I diagnosed the tumor to be a fibro-sarcoma of the right ovary because we very rarely find such a dropsy associated with such an ovarian tumor, without the tumor being malignant.

At 2 P.M., with strict antiseptic precautions, I made an incision of 2 inches into the abdominal cavity, below the navel. The peritonitic fluid was discharged (about 5 gallons), and the solid, gray, hard tumor presented at the opening. Two fingers introduced, found that it grew from the right ovary—free from attachments—except abdominal at one point. Much oozing of blood from abdominal incision, and although hæmostatic forceps were used in great number, yet the oozing was troublesome. Incision enlarged to 4 inches, but I was unable to get the tumor out till I had enlarged the opening from navel to pubis. Abdominal adhesions were then torn away, and as they bled freely, were gathered up and tied. The pedicle was transfixed and tied on either side, with silk; and one of the ligatures was brought entirely around the whole pedicle, and tied again, when the tumor was cut away. Abdominal cavity was thoroughly sponged and all bleeding checked. A drainage tube was inserted, and the abdominal opening closed with 12 silk sutures.

Wound dressed with iodoform and borated cotton-sponge over drainage tube, wrapped in rubber cloth and the whole covered with a loose flannel bandage.

She was put to bed—surrounded with bottles of hot water, and wrapped in blankets. Reaction came on slowly. No nausea. She returned in due time to perfect consciousness. Pulse very frequent and feeble.

September 4th.—Temperature 100. It rose once to $101\frac{3}{5}$, but never above this; and was most of the time below 100. Pulse 130, small and feeble. Pulse continued to rise and temperature to fall, till her death on the third day. She slept several hours on 25 gtts of deod. tinct. opium, during first night—given by the rectum. This had to be repeated several times during the first 36 hours on account of pain, but always quieted.

She was given milk, brandy and hot water, and digitalis by the mouth, brandy and milk by the rectum, and quinine, hypodermically, but nothing seemed to bring on perfect reaction, and she died in 67 hours after the operation.

She evidently died of shock. I am at a loss to know why the shock was so great and uncontrollable. The malignancy of the tumor, and more or less poisoned condition of blood, must have had something to do with it—but how? The sudden removal of such an enormous distention of the abdomen, was also an important factor in the shock.

I am also at a loss to know why such a tumor, not pressing on any important organs or vessels, could have produced such a dropsy. Its malignant nature was the cause—but how?

I herewith submit the report of Professor Welch, of the Johns Hopkins Hospital on the character of the tumor:

*My Dear Dr. Wilson:* I send the report of my examination of the interesting tumor which you sent me recently. The tumor is unquestionably of ovarian origin and is, as you surmised, a fibro-sarcoma. The extensive death by necrosis of so large a part of the tumor is an interesting feature.

The specimen is a solid tumor, nearly spherical in shape, measuring 15 ctm. in diameter.

The fimbriated extremity of the fallopian tube, to the extent of 6 ctm., has been removed and is connected with the tumor by a part of the broad ligament (mesovarium) measuring 3 ctm. in width.

The tumor is solid with the exception of two small cysts containing fluid blood contents, near the attachment of the mesovarium. There is separable peritoneal covering of the tumor. Upon section the tumor presents an outer zone, varying from 3 to 5 ctm. in thickness, of a grayish homogeneous appearance, and a central mass of an opaque yellowish-white appearance. This central mass is succulent, œdematous.

Upon microscopical examination the outer zone of the tumor is found to be composed of parallel bundles of connective tissue, which in places is replaced by closely packed fusiform cells. Toward the central mass of the tumor, the outer zone is infiltrated richly with round small cells, accumulated chiefly around small veins and capillaries. Many blood-vessels in this peripheral zone are the seat of an obliterating endarteritis.

The central yellowish-white, œdematous mass which makes up the greater bulk of the tumor is chiefly necrotic, as is shown by the absence of nuclear staining, although the tumor was received in good condition, and fresh pieces were at once hardened in strong alcohol. The contours of abundant round and fusiform cells, devoid of stained nuclei, can be seen on sections through this part of the tumor. The walls of blood-vessels are entensively calcified in this region. There are many large spherical, oval and irregular cells filled with coarse, fatty globules, resembling fatty granular cells. The connective tissue fibres are split up into fine, interlacing fibrillæ.

No epithelial lining can be found in the two small cysts mentioned above.

The tumor is evidently an ovarian tumor. It has not the relations to the fallopian tube of an intraligamentous new growth.

Its microscopical structure is that of a fibro-sarcoma. The central and greater part of the tumor has undergone coagulation-necrosis, and partial calcification, doubtless as the result of imperfect nutrition from disease of the blood-vessels.

*Diagnosis.*—Fibro-Sarcoma Ovarii.

Dr. Wm. P. Chunn would prefer chloroform to ether when the patient was excitable and where the operation promised to be short. So much less chloroform being necessary for anæsthesia than ether. It would seem better always to give four drachms of chloroform than a pint of ether. In regard to ascites, caused by solid tumors in the abdomen, at first sight, it seemed strange that a *small* solid growth would produce such an accumulation of fluid, while a much larger *cyst* may cause no secretion whatever. It may be explained in the following way. The solid tumors, being malignant, irritate the peritoneum which results in hypersecretion and consequently ascites.

Dr. Robert T. Wilson: After the operation I was informed by the anæsthetist that less than two ounces of chloroform was used.

Dr. L. E. Neale thought that in addition to the effect of chloroform, hæmorrhage, etc., the sudden removal of intra-abdominal pressure consequent upon the rapid withdrawal of so large a quantity of ascitic fluid might have been a factor in the causation of heart failure, shock and death.

Time or duration of operation must have been another potent factor, for Dr. Wilson had omitted to explain why the removal of such a slightly adherent tumor had occupied one hour and a half.

In connection with this case he would state that although the presence of large or rapidly accumulating ascites with an abdominal tumor was usually regarded as an indication of malignancy of the tumor, on September 18th, 1886, he had removed three gallons of such fluid during an operation for the removal of the uterine appendages for interstitial uterine fibroid in a negress.

The result was successful, and while the tumor gradually wasted away, the fluid never returned.

